# Unfolding the policy dynamics of medical data assetization in Chinese public healthcare institutions: evidence from Latent Dirichlet Allocation and dynamic topic modeling analysis

**DOI:** 10.3389/fpubh.2026.1805248

**Published:** 2026-04-20

**Authors:** Su Han, Qihang Zeng, Xinyu Cui, Yuanyi Ji, Kexin Liu, Nan Li, Yongzhong Cheng

**Affiliations:** 1 Department of Operation and Management, West China School of Public Health and West China Fourth Hospital, Sichuan University, Chengdu, Sichuan, China; 2 Department of Health Policy and Management, West China School of Public Health and West China Fourth Hospital, Sichuan University, Chengdu, Sichuan, China; 3 Discipline Inspection Commission Office, West China Hospital, Sichuan University, Chengdu, Sichuan, China; 4 Hospital-Acquired Infection Control Department, West China School of Public Health and West China Fourth Hospital, Sichuan University, Chengdu, Sichuan, China; 5 Information Center, West China Hospital, Sichuan University, Chengdu, Sichuan, China; 6 Party Affairs Office, West China Hospital, Sichuan University, Chengdu, Sichuan, China

**Keywords:** China, dynamic topic model, LDA, medical data assets, policy evolution, public health governance

## Abstract

**Background:**

As medical data increasingly functions as a factor of production, public healthcare systems face the challenge of reconciling data-driven innovation with regulatory accountability. In China, a series of national initiatives has sought to promote the assetization of medical data through market-oriented mechanisms. However, existing studies have primarily focused on legal frameworks, technical architectures, and ownership debates, while the temporal evolution of policy priorities and the interaction between central design and local implementation remain insufficiently understood.

**Methods:**

This study constructs a corpus of 192 policy documents (47 national and 145 provincial) issued between 2015 and 2025 and applies Latent Dirichlet Allocation (LDA) and Dynamic Topic Modeling (DTM) to examine both the structural composition and temporal evolution of policy agendas. Specifically, LDA is used to identify latent thematic structures at national and provincial levels, while DTM traces shifts in topic intensity across policy phases and captures the dynamic relationship between central agenda-setting and provincial policy activation.

**Results:**

The results reveal a clear differentiation in governance focus. National policies emphasize macro-level institutional design, security governance, and authorized operation, with valuation-related discourse exhibiting a short-term surge followed by a strategic reorientation toward operational feasibility. Provincial policies, by contrast, display a more implementation-oriented trajectory, progressively prioritizing registration procedures, circulation mechanisms, and trading infrastructure. Dynamic analysis further shows that policy diffusion follows a staged pattern rather than linear synchronization, with 2023 marking a critical inflection point for local policy activation.

**Conclusion:**

Together, the findings indicate a functional division of policy labor between central and local governments. Medical data assetization in China is advancing through a cautious and adaptive governance process in which authorization, registration, and secure circulation precede stable valuation. This study provides empirical evidence on the sequencing logic of medical data assetization and demonstrates the value of computational policy analysis for understanding multi-level governance dynamics in public health data governance.

## 1 Introduction

The digitalization of healthcare systems has generated an unprecedented volume of medical data, transforming it from a mere by-product of clinical care into a strategic factor of production. Globally, integrating health data into the economic cycle while safeguarding public interest remains a formidable governance challenge. While frameworks like the GDPR (1) in Europe and HIPAA (2) in the United States focus primarily on privacy and security boundaries, China presents a distinct governance experiment, in which the state has attempted to transform medical data from “resources” into market-oriented “assets” through an evolving mix of development, governance, and regulatory policies (3, 4). Since the inclusion of data as a factor of production in 2020 (5), China has accelerated the construction of a data element market, attempting to reconcile the public attributes of healthcare with the efficiency of market allocation.

For Public Healthcare Institutions (PHIs), which serve as the principal holders of high-quality clinical data in China, this transition marks a profound shift in operational logic. Unlike the early phase of “Informatization” (2010–2015), where the goal was establishing Electronic Medical Records (EMR) for internal efficiency (6), the current phase of “Assetization” (2020–present) demands that PHIs engage in data confirmation, valuation, and authorized trading (7). This shift is driven by a series of high-level policies, culminating in the “Data Element X Action Plan (2024–2026)” (8) and the “Interim Provisions on Accounting Treatment of Enterprise Data Resources” (9). These policies are intended to activate the economic and social value of medical data, including its potential applications in pharmaceutical R&D, commercial insurance innovation, and AI diagnostics (3, 6, 10, 11).

Despite the ambitious top-level design, the implementation of medical data assetization continues to face the dilemma of “Rich Data, Poor Value” (12), in which abundant data resources do not automatically translate into stable value realization (3, 6, 9, 10). In practice, PHIs often adopt a conservative stance due to ambiguous ownership definitions, lack of standardized pricing mechanisms, and compliance risks. This disconnect suggests a potential misalignment between the central government’s strategic intent (macro-level value creation) and local governments’ execution pathways (micro-level implementation). Understanding the evolutionary trajectory of policy supply is crucial to diagnosing this bottleneck. It requires dissecting how policy instruments have shifted over time—from encouraging infrastructure connectivity to establishing complex trading rules—and whether local policy responses have synchronized with central directives.

Existing literature on medical data governance has largely focused on three dimensions: legal frameworks for privacy protection (11, 13), technical architectures for secure data sharing (e.g., blockchain and federated learning) (11, 14), and theoretical debates on data ownership rights (15). While these studies provide valuable static insights, there is a paucity of quantitative empirical research on the policy evolution itself (13, 16).

First, most policy analyses are qualitative or focus on single regulations, failing to capture the temporal granularity of policy themes over the past decade. The evolution from “digital resource management” to “asset valuation” involves subtle shifts in keyword frequency and topic intensity that manual reading cannot fully quantify.

Second, the spatial dimension of policy diffusion remains underexamined. In China’s unitary yet decentralized administrative system, provincial governments often act as key filters and translators of central policies (17, 18), especially in policy domains that require both regulatory compliance and local procedural adaptation. Current research has yet to systematically measure the consistency, lag, or heterogeneity between national directives and provincial implementation using computational methods.

Recent empirical studies have begun to quantify adjacent policy domains and thus provide an important point of departure for the present research (4, 19–21). Quantitative analyses of China’s health and medical big data policies have revealed variation in thematic coverage, policy-tool combinations, and policy effectiveness across central and provincial texts, suggesting that this field is better understood as a multi-level governance structure than as a uniform policy regime. Related studies on data-element policy evolution, smart-city policy trajectories, and provincial public data management rules further indicate that China’s digital governance agenda is characterized by stage-based adjustment, regional differentiation, and the gradual refinement of institutional rules. However, these studies have largely focused on health big data, general data-element governance, or public data management in adjacent contexts. They have not yet examined, in a systematic and dynamic way, how policy priorities evolve specifically in the field of medical data assetization, how national and provincial agendas diverge in thematic emphasis, or how their relationship unfolds over time in terms of diffusion, lag, and functional differentiation. The present study addresses this gap by moving the analysis to the intersection of medical data assetization, central–provincial comparison, and dynamic policy evolution.

Compared with prior studies that primarily examine medical data governance through legal, technical, or ownership-based lenses, the present study shifts attention to policy evolution as an empirical and comparative problem. Existing international research has provided important insights into privacy protection, data sharing architectures, and governance frameworks for health data (11, 13–16) but has generally not addressed how policy agendas evolve over time within a multi-level state structure. Recent Chinese studies have begun to quantify adjacent domains, including health and medical big data policy, data-element policy evolution, and provincial public data management (4, 19–21). However, these studies either focus on broader data governance contexts or remain limited to static policy-text descriptions. They do not systematically examine how medical data assetization, as a more specific and institutionally sensitive policy domain, develops through the interaction between national agenda-setting and provincial implementation. By combining LDA and DTM, this study contributes to the literature in three respects: it provides a dynamic empirical account of policy evolution in medical data assetization; it clarifies the differentiated roles of central and provincial governments in this process; and it extends existing policy-text research from static thematic mapping to the analysis of temporal change and multi-level governance dynamics.

To bridge these gaps, this study employs a computational social science approach to reconstruct the policy landscape of medical data assetization in China. We curated a comprehensive corpus of 192 policy documents (47 national and 145 provincial) issued between January 2015 and June 2025. By utilizing Latent Dirichlet Allocation (LDA) (22) and the Dynamic Topic Model (DTM) (22), this study examines medical data assetization as a process of institutional change embedded in multi-level data governance. From this perspective, it is necessary to attend not only to what policy themes are present, but also to how they evolve over time and how they are differentiated across levels of government. Accordingly, this study addresses the following research questions: (i) What are the dominant thematic structures of national and provincial medical data assetization policies in China? (ii) How have these policy priorities evolved over time, particularly across key phases of institutional development? (iii) How does the relationship between central policy design and provincial implementation unfold in terms of thematic divergence, temporal lag, and functional differentiation? To answer these questions, we pursue three interrelated objectives: (i) to identify the latent topic structures of national and provincial policies, thereby clarifying their respective governance priorities; (ii) to trace the continuous evolutionary paths of policy themes, with particular attention to the changing salience of asset valuation, authorized operation, and market circulation across policy phases; and (iii) to reveal the dynamic relationship between central design and local execution, thereby illuminating the multi-level governance dynamics of medical data assetization. This study provides timely, data-driven evidence on how a major public health system navigates the complex transition from digital governance to data assetization.

### 1.1 Conceptual framework

In this study, medical data assetization refers to the process by which medical data are gradually incorporated into institutional arrangements that enable their registration, authorized use, circulation, and potential valuation under public governance. It should therefore be understood not simply as market trading, but as a broader governance process through which medical data are transformed from administrative resources into governable and economically usable assets. Within this framework, central policy design refers to the national-level articulation of strategic direction, legitimacy boundaries, and basic institutional rules, whereas provincial implementation refers to the process through which local governments translate these broad principles into operational procedures, platform arrangements, and regulatory routines (11, 13, 16).

The analytical logic of this study rests on the assumption that medical data assetization is shaped by a multi-level governance process rather than by a single linear policy command. National policies are expected to define the permissible scope and institutional conditions of data use, while provincial policies are expected to operationalize these conditions through concrete mechanisms. Among these mechanisms, authorized operation and registration are treated as foundational governance instruments because they clarify usage conditions, responsibility chains, and procedural legitimacy. Valuation, by contrast, is understood as a more advanced and institutionally demanding stage that depends on the prior stabilization of rights arrangements, circulation mechanisms, and compliance infrastructures. From this perspective, divergence between central and provincial policies is not necessarily a sign of inconsistency; it may instead reflect a functional differentiation between strategic boundary-setting and procedural implementation (4, 17, 18, 23).

## 2 Materials and methods

### 2.1 Data collection and preprocessing

To construct a representative corpus of China’s medical data assetization policies, we retrieved documents from the authoritative PKULaw database (Peking University Law Database) and official government portals, covering the period from January 1, 2015, to June 30, 2025. The search strategy employed a combination of keywords encompassing core subjects (e.g., “Medical Data,” “Health Big Data,” “Clinical Data”) and functional processes (e.g., “Assetization,” “Valuation,” “Trading,” “Authorized Operation”). This study adopted a document-based sampling design aimed at capturing both top-level institutional design and provincial implementation within the evolving policy field of medical data assetization.

We adopted a hierarchical inclusion strategy to capture the full policy ecosystem. The selection criteria retained: (i) macro-level laws, regulations, and plans governing the data element market and digital economy, which establish the fundamental institutional framework and accounting standards applicable to public sectors (including Public Healthcare Institutions); and (ii) domain-specific normative documents explicitly addressing medical data governance, health big data applications, and industry-specific digitalization. We excluded purely technical standards unrelated to governance rules and low-substance administrative replies. After retrieval, duplicated and irrelevant records were manually screened according to these predefined inclusion and exclusion criteria, and only documents with substantive policy relevance to medical data assetization were retained in the final corpus.

This process yielded a final dataset of 192 valid policy documents, consisting of 47 national-level and 145 provincial-level policies. The final sample therefore captures both central policy design and provincial implementation, and includes multiple document types such as laws, regulations, notices, plans, and implementation measures. The complete list of the policy documents analyzed in this study is provided in the Supplementary material.

Prior to modeling, standard natural language processing (NLP) techniques were applied. We used the *Jieba* library (24) for Chinese text segmentation, augmented with a user-defined dictionary containing domain-specific terms such as “Data Element” and “Asset Valuation” to ensure segmentation accuracy. A composite stop-word list was then utilized to filter out noise, followed by Term Frequency-Inverse Document Frequency (TF-IDF) (25) calculation to identify and retain words with high discriminatory power for subsequent modeling.

### 2.2 Latent Dirichlet Allocation (LDA) topic modeling

We employed the Latent Dirichlet Allocation (LDA) model, a generative probabilistic framework, to uncover the latent semantic structures within the policy text. LDA assumes that each document is a mixture of topics and that each topic is a mixture of words. In the present study, this analytical strategy is based on the assumption that policy documents contain latent thematic mixtures that can be inferred from recurring word co-occurrence patterns. Although the bag-of-words representation does not preserve word order, it is appropriate for identifying broad thematic structures in a large policy corpus, where the primary objective is to detect stable patterns of semantic emphasis rather than sentence-level meaning (22).

To determine the optimal number of topics (K)—a critical parameter for model interpretability—we calculated Perplexity and Coherence Scores (26) across a range of K values from 2 to 15. The results indicated that the model achieved optimal performance at K = 8 for the National Corpus and K = 4 for the Provincial Corpus, balancing mathematical coherence with semantic distinctiveness. The hyperparameters *α* (document-topic density) and *β* (topic-word density) were set to auto-optimization modes to fit the data distribution best. This static modeling phase allowed us to identify the foundational thematic architecture of governance priorities at both central and local levels. Accordingly, the first stage of the analysis focused on extracting and interpreting the static topic structures of the national and provincial corpora separately, so as to establish a baseline for subsequent dynamic comparison.

### 2.3 Dynamic topic model (DTM) analysis

While standard LDA provides a static snapshot, it fails to capture the temporal evolution of policy focus. To address this limitation, we implemented the Dynamic Topic Model (DTM) based on variational Kalman filtering (27). DTM extends LDA by assuming that the topic distribution and word probabilities in a current time slice (t) evolve from the previous slice (t − 1), thereby enabling the tracking of thematic intensity over time. This approach is based on the assumption that policy agendas change in a temporally dependent rather than fully discontinuous manner, making it possible to trace gradual shifts in thematic emphasis across adjacent periods. In other words, the model assumes that topic evolution is path-dependent: later policy priorities are shaped by, rather than fully detached from, earlier institutional arrangements and policy signals (27).

Following the coding scheme in our methodological notes, we constructed time slices based on policy issuance periods and aggregated documents into three policy phases: a foundational phase (2015–2022), a systematic construction phase (2023–2024), and a precision landing phase (2025). This phase-based slicing balances temporal interpretability with the practical constraint of uneven yearly document counts, enabling the model to identify major agenda shifts associated with key institutional milestones. The decision to adopt phase-based rather than year-by-year slicing also reflects the assumption that major institutional transitions are more analytically meaningful than short-term annual fluctuations in a corpus with uneven document frequency.

The second stage of the analysis therefore applied DTM separately to the national and provincial corpora in order to trace how topic salience changed across policy phases and to compare the dynamic relationship between central policy design and provincial implementation. Topic labelling was conducted as an interpretive step after model estimation rather than as an automatic output of the DTM. Specifically, labels were assigned by jointly considering (i) the highest-probability keywords associated with each topic across time slices, (ii) the semantic consistency of these keywords over time, and (iii) representative policy documents with high topic proportions. To reduce subjective bias, two researchers independently reviewed the keyword sets and proposed candidate labels, and any discrepancies were resolved through discussion with a third senior researcher until consensus was reached. Labels were retained only when they were consistently supported by both the keyword structure and the substantive content of the corresponding policy texts. These labels are intended to facilitate substantive interpretation of the model outputs and do not alter the underlying probabilistic structure of the DTM.

## 3 Results

### 3.1 Descriptive analysis of policy supply

The policy corpus analyzed in this study spans the period from January 2015 to June 2025 and covers both national-level directives and provincial implementation plans. The statistical analysis of these documents reveals specific patterns in the timing of policy issuance and geographic distribution.

#### 3.1.1 Temporal trends in policy frequency

**Figure 1:**
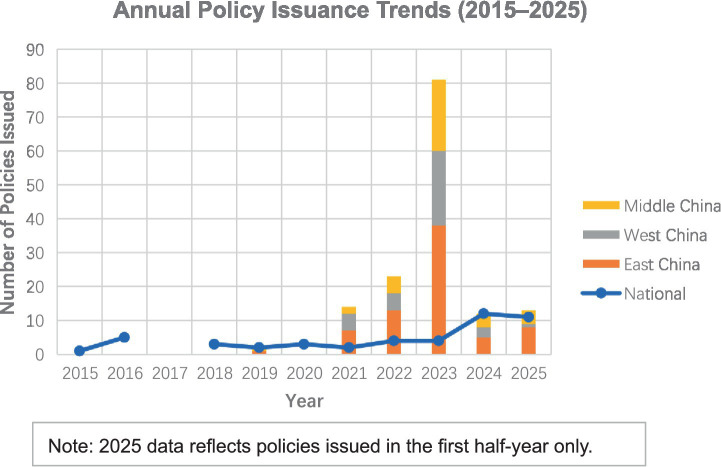
Spatiotemporal distribution of medical data assetization policies in China (2015–2025). The stacked bars represent the number of provincial policies issued annually, categorized by region (Eastern, Central, and Western). The chart illustrates a significant surge in policy issuance in 2023 following the national “Twenty Measures on Data”.

The longitudinal data indicates a non-linear growth trajectory in policy supply (Figure 1). Between 2015 and 2020, the annual volume of policies remained low, corresponding to the initial phase of medical data management. A noticeable increase occurred in 2021, with 14 provincial policies issued, following the central government’s designation of data as a factor of production.

The most significant finding is the sharp peak in 2023. As illustrated in Figure 1, provincial policy issuance exhibits a sharp surge in 2023, accounting for more than half of the total local policy output. This concentrated volume aligns with the release of the “Twenty Measures on Data” in late 2022, suggesting not merely a rapid administrative response at the local level, but a broader institutional inflection point at which national agenda-setting was translated into large-scale provincial policy activation. Following this surge, the volume moderated to 12 documents in 2024 and 13 in the first half of 2025. Rather than indicating a decline in policy attention, this reduction in quantity appears to coincide with a shift from broad agenda mobilization toward more targeted and operational policy instruments, reflecting a transition from policy expansion to implementation refinement.

#### 3.1.2 Regional distribution characteristics

The spatial analysis of the 145 provincial documents demonstrates an uneven distribution across economic zones. The Eastern region contributes the largest share with 72 documents (49.7%), consistent with the concentration of digital economy infrastructure in provinces such as Zhejiang and Guangdong.

However, the policy activity in the Western and Central regions shows a balanced pattern that differs from typical economic gradients. The Central region accounts for 37 documents (25.5%), while the Western region follows closely with 36 documents (24.8%). Despite a lower economic baseline, the Western region maintains a high level of policy output. This is largely attributable to the “East Data, West Computing” strategic initiative (21), which has prompted provinces like Guizhou and Sichuan to accelerate their regulatory frameworks for data resource management. More broadly, this pattern suggests that medical data assetization is not being driven solely by the spontaneous diffusion of market incentives from economically advanced regions; rather, it is being shaped by a nationally coordinated strategic agenda that has activated policy responses across different regional development contexts.

### 3.2 Static thematic landscape of policy documents

The Latent Dirichlet Allocation (LDA) model identified distinct latent semantic structures within the policy corpus, revealing a divergence in governance priorities between central and local administrations.

#### 3.2.1 National policy themes

For the National Corpus, the optimal topic number was determined at K = 8. As detailed in Table 1, the central government’s governance priorities can be categorized into three functional clusters.

**Table 1:** Latent topics and high-frequency keywords of national policies (K = 8).

Topic no.	Probability (%)	Topic label	Top 10 keywords
Topic 1	26.5%	Digital Infrastructure and Economy	Trusted Data Space, Digital Economy, Circulation, Data Resource, Infrastructure, Collaboration, Data Element, Digital, Data Circulation, Entity
Topic 2	19.9%	Authorized Operation and Public Data	Public Data, Registration, Authorized Operation, Authorization, Public, Development and Utilization, Data Security, Responsibility, Data Resource, Opening
Topic 3	13.8%	Macro Strategy and Governance	Economy, Culture, Strategy, International, Reform, Leadership, Safeguard, Optimization, Politics, Governance
Topic 4	13.6%	Informatization Legacy	Big Data, Informatization, Network, Internet, Information, Industry, Platform, International, Digitization, R&D
Topic 5	7.9%	Market Allocation Mechanism	Element, Reform, Unified, Marketization, Pilot, Supervision, Allocation, Trading, Region, Orderly
Topic 6	7.1%	Data Asset Valuation	Data Asset, Value, Yield, Data Resource, Asset, Rights, Asset Valuation, Intangible Asset, Prediction, Business
Topic 7	6.3%	Healthcare Domain Specifics	Health, Health Big Data, Medical, Healthcare, Monitoring, TCM, Drugs, Internet, NHC, Medical Institution
Topic 8	5.0%	Legal Liability and Security	Network, Network Security, Data Security, Operator, Information, Fine, Law, In accordance with law, CII, Regulation

The first cluster represents the Strategic Foundation. Topic 1 (Digital Infrastructure and Economy) dominates with the highest probability (26.5%). Unlike early-stage infrastructure policies, this topic is characterized by the keyword “Trusted Data Space” (28), which ranks first. This indicates a paradigm shift where the state prioritizes building secure, trusted technological environments to facilitate data circulation. Topic 3 (Macro Strategy and Governance) (13.8%) provides the overarching context, linking data governance to broader national goals such as “Economy,” “Culture,” and “Strategy.” Topic 4 (Informatization Legacy) (13.6%) reflects the continuity of historical policies, focusing on “Big Data” and “Platform” construction.

The second cluster addresses the Institutional Supply for Assetization. Topic 2 (Authorized Operation and Public Data) (19.9%) establishes the primary mechanism for separating data ownership from usage rights, featuring terms like “Authorized Operation” and “Registration.” Crucially, Topic 6 (Data Asset Valuation) (7.1%) marks the transition to financialization. High-frequency keywords such as “Intangible Asset,” “Yield,” and “Asset Valuation” provide empirical evidence that national policies are now explicitly addressing the accounting recognition and pricing of data assets. Topic 5 (Market Allocation Mechanism) (7.9%) complements this by outlining the “Marketization” and “Unified” rules for factor allocation.

The third cluster defines the Security Boundaries. Topic 8 (Legal Liability and Security) (5.0%) functions as the regulatory constraint. Dominated by strict legal terms like “Fine,” “Law,” and “CII” (Critical Information Infrastructure), this topic delineates the red lines for data operators, ensuring that economic exploitation does not compromise national security.

#### 3.2.2 Provincial policy themes

In contrast to the macro-institutional design at the national level, the Provincial Corpus (K = 4) exhibits a highly pragmatic and execution-oriented structure. As summarized in Table 2, the governance priorities at the local level are concentrated on industrial application and market operations.

**Table 2:** Latent topics and high-frequency keywords of provincial policies (K = 4).

Topic no.	Probability (%)	Topic label	Top 10 keywords
Topic 1	33.8%	Digital Economy Transformation	Digital Economy, Digitization, Innovation, Manufacturing, Transformation, Industry, Enterprise, Industrial Structure, Development, Integration
Topic 2	26.6%	Data Registration and Compliance	Registration, Certificate, Applicant, Change, Public Data, Review, Agency, Compliance, Catalog, Right Holder
Topic 3	24.3%	Market Trading and Circulation	Trading, Exchange, Data Vendor, Circulation, Profit Distribution, Brokerage, Market, Cross-border, Service Provider, Ecosystem
Topic 4	15.3%	Digital Gov and Public Services	Government Data, E-License, Government Cloud, Sharing, Collaboration, Service, Unified, Responsibility, Efficiency, Online

Topic 1 (Digital Economy Transformation) accounts for the largest share (33.8%). Unlike the national focus on “Infrastructure,” local policies prioritize the outcomes of digitization, with keywords like “Manufacturing,” “Enterprise,” and “Industrial Structure.” This suggests that provincial governments view medical data assetization primarily as a lever to drive regional economic growth and industrial upgrading.

Topic 2 (Data Registration and Compliance) (26.6%) represents the “first mile” of local implementation. While national policies define the abstract right to operate, provincial policies detail the bureaucratic workflow. The high frequency of procedural keywords such as “Certificate,” “Applicant,” and “Review” indicates that local governments are actively constructing a “Data Property Rights Registration System,” issuing official certificates to formalize data ownership.

Topic 3 (Market Trading and Circulation) (24.3%) corresponds to the operationalization of the market. This topic features specific market entities and rules that are less prominent in national documents. Notably, terms like “Data Vendor” and “Profit Distribution” appear frequently, revealing that provinces are pioneering the exploration of “who trades” and “how to distribute value.”

Topic 4 (Digital Gov and Public Services) (15.3%) focuses on internal administrative efficiency. Keywords like “E-License” and “Government Cloud” reflect the effort to break down data silos between government departments (e.g., Health Commission and Medical Insurance Bureau) to facilitate seamless data sharing.

Taken together, these static topic structures suggest that, compared with the national emphasis on macro-institutional design, provincial governments are more directly concerned with proceduralizing data rights, circulation, and market operation in administratively workable forms.

### 3.3 Evolutionary trajectory of policy priorities

The Dynamic Topic Model (DTM) results reveal a non-linear evolutionary trajectory for both national and provincial policies. Unlike a simple linear progression, the data exposes distinct “policy cycles,” characterized by rapid thematic shifts in response to implementation challenges.

#### 3.3.1 Evolution of national policies

To capture the temporal evolution of policy priorities, the Dynamic Topic Model (DTM) was applied to the national policy corpus (K = 8). It is worth noting that the DTM analysis operates independently of the static LDA model. Therefore, the topic indices in the DTM visualizations and subsequent text (starting from Topic 0) do not directly correspond to the topic numbers (starting from Topic 1) discussed in Section 3.2. Following the labelling procedure described in Section 2.3, the dynamic topics were interpreted on the basis of the highest-probability keywords across time slices, their semantic consistency over time, and representative policy documents with high topic proportions. The resulting topics are labeled as follows: Topic 0 (Info-Tech Standardization), characterized by *‘technology’* and *‘standard’*; Topic 1 (Macro Strategy), focusing on *‘system’* and *‘governance’*; Topic 2 (Data Security), highlighted by *‘network security’* and *‘infrastructure’*; Topic 3 (Healthcare Regulation), specific to *‘medical’* and *‘health’*; Topic 4 (Authorized Operation), centered on *‘public data’* and *‘authorization’*; Topic 5 (Market Allocation), emphasizing *‘element’* and *‘transaction’*; Topic 6 (Digital Economy), defined by *‘industry’* and *‘innovation’*; and notably, Topic 7 (Asset Valuation), characterized by distinct keywords such as *‘asset’*, *‘value’*, *‘yield’*, and *‘pricing’*.

**Figure 2:**
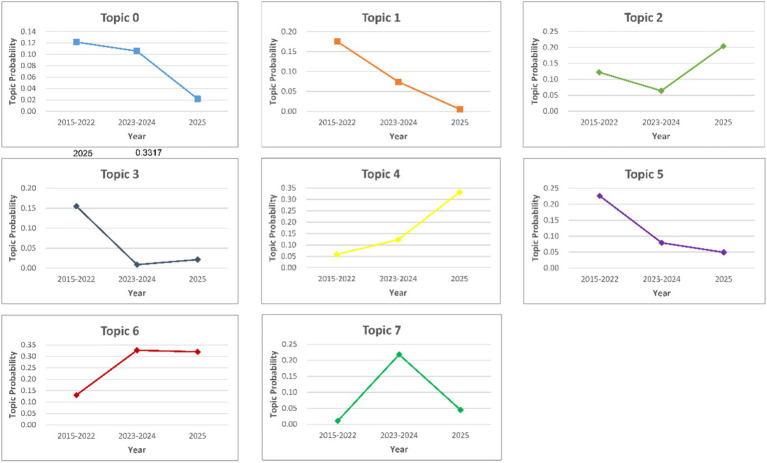
Evolutionary trajectory of national policy priorities (2015–2025).

The evolutionary trajectory of these topics, visualized in Figure 2, demonstrates a distinct transition from theoretical framework construction to mechanism implementation. In the period leading up to 2022, the policy landscape was characterized by a relatively balanced distribution of topics, with Topic 5 (Market Allocation) and Topic 1 (Macro Strategy) maintaining intensities around 0.20. This phase reflected the initial establishment of the data element market’s institutional foundation.

A significant shift occurred during 2023 and 2024, where Topic 7 (Asset Valuation) emerged as a focal point, reaching an intensity peak of 0.218. This trend coincided with the release of the “Twenty Measures on Data,” which prioritized the exploration of pricing mechanisms and asset accounting. However, the data for 2025 indicates a notable strategic adjustment. The intensity of Topic 7 decreased to 0.045, while Topic 4 (Authorized Operation) rose substantially to 0.332, becoming the dominant theme. Concurrently, Topic 2 (Data Security) increased to 0.204. This reversal suggests that recent national policies have reoriented towards the practical “Authorized Operation” mechanisms and technical infrastructure (e.g., trusted data spaces) required to support data circulation, rather than focusing solely on valuation methodologies.

#### 3.3.2 Evolution of provincial policies

Following the national-level analysis, the Dynamic Topic Model (DTM) was further applied to the provincial policy corpus using the optimal topic number identified by the static LDA analysis (K = 4). The four dynamic topics generated by the model jointly delineate the core agenda of local data governance and its evolution over time. In line with the labelling procedure described in Section 2.3, and based on keyword composition, semantic orientation, and representative policy documents with high topic proportions, these topics are identified as: Topic 0 (Public Data Assetization and Market Circulation), Topic 1 (Digital Economy and Industrial Innovation), Topic 2 (Digital Government and Administrative Governance), and Topic 3 (Data Asset Registration and Rights Confirmation).

**Figure 3:**
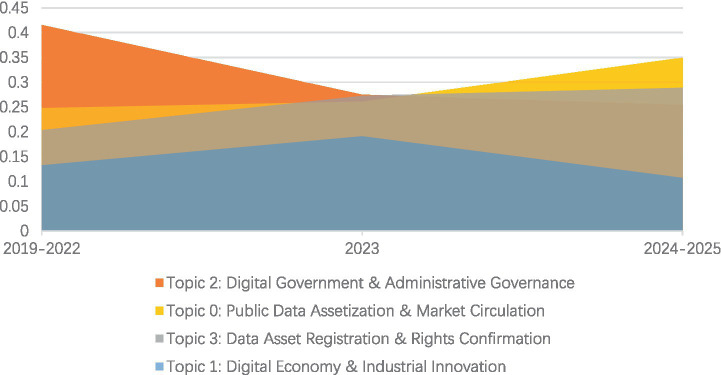
Temporal dynamics of provincial policy themes (2019–2025).

As shown in Figure 3, provincial policy evolution exhibits a clear stage-based transition from internal governance to external market activation. In the initial phase (2019–2022), Digital Government dominated the policy agenda, with the highest topic intensity (0.415). Keywords such as “government affairs,” “platform,” and “system” indicate that local governments primarily focused on building digital administrative capacity and governance infrastructure during this period.

A turning point emerged in 2023, when the intensity of Digital Government began to decline, while Public Data Assetization and Digital Economy topics increased simultaneously. The rising prominence of terms such as “transaction,” “factor,” “enterprise,” and “authorization” suggests that provincial policies started to extend beyond internal digitization toward market-oriented exploration under evolving national frameworks.

In the most recent phase (2024–2025), the policy agenda shifted decisively. Public Data Assetization became the dominant theme (0.349), accompanied by a sustained rise in Data Asset Registration and Rights Confirmation (0.289). The concentration of keywords such as “registration,” “authorized operation,” “intellectual property,” and “asset” indicates that local governments are prioritizing the formalization of data rights and the institutionalization of data circulation mechanisms.

### 3.4 Spatiotemporal disparity analysis

A comparative analysis of national and provincial policy trajectories reveals both temporal lag and spatial heterogeneity in the implementation of medical data assetization policies.

#### 3.4.1 Temporal lag and policy diffusion

The diffusion of national policy agendas into local implementation exhibits a clear stage-based pattern rather than a linear progression. Although market-oriented concepts related to data elements appeared sporadically in provincial policies during 2019–2022, policy activity at the local level remained limited in scale during this period. This phase corresponds to an institutional preparation stage, in which local governments focused primarily on internal governance capacity building.

A decisive shift occurred in 2023, when provincial policy issuance increased sharply across all regions. The concentration of policy outputs in this single year indicates that local governments began to respond collectively once national-level frameworks regarding public data governance and authorized operation became more explicit. Rather than immediate synchronization with central agenda-setting, local implementation followed a “threshold response” pattern, in which substantive policy expansion was triggered only after key institutional boundaries were clarified.

This temporal structure is consistent with the DTM results, which show that themes related to public data circulation and asset registration intensified markedly after the preparatory phase. Together, these findings suggest that the central–local relationship in medical data assetization is characterized less by continuous lag than by staged diffusion, with 2023 serving as a critical inflection point for local policy activation. This implies that local implementation is triggered not by immediate one-to-one synchronization with central directives, but by the clarification of institutional boundaries that make large-scale procedural translation feasible.

#### 3.4.2 Regional heterogeneity

From a spatial perspective, the distribution of provincial policies demonstrates a pattern of “synchronized mobilization with differentiated focus.” Eastern provinces account for the largest share of policy outputs, reflecting their stronger digital economy foundations and market capacity. However, the timing of this mobilization reveals strong national coordination: the simultaneous rise in policy activity across Central and Western regions in 2023 mirrors the trend in the East. This indicates that medical data assetization is not confined to economically advanced areas but is a nationally orchestrated campaign triggering a unified response.

Despite this temporal synchronization, the substantive focus diverges regionally. In particular, while maintaining high policy intensity, Western and Central provinces prioritize different governance themes. While eastern provinces focus more on market mechanisms and application scenarios, central and western regions place greater weight on infrastructure construction, data security, and institutional frameworks, often under national strategic initiatives such as “East Data, West Computing.”

This pattern reflects a differentiated but complementary spatial structure. Economically advanced regions lead in market experimentation, while strategically supported regions contribute to resource aggregation and system-level stability. Together, they form a multi-regional implementation landscape, although this heterogeneity also raises challenges for cross-regional coordination and policy interoperability. In this sense, the diffusion of medical data assetization in China is best understood as a centrally framed but regionally differentiated process, rather than as either a uniform administrative rollout or a purely market-driven expansion.

## 4 Discussion

### 4.1 A non-linear and corrective evolution of national policy priorities

The national-level policy trajectory of medical data assetization does not conform to a linear progression from informatization to marketization. Instead, the Dynamic Topic Model (DTM) results indicate a pattern of iterative adjustment, in which periods of agenda acceleration are followed by corrective reorientation. This non-linear evolution reflects the inherent tension between policy ambition and institutional feasibility in governing sensitive public-sector data.

During the initial phase from 2015 to 2022, national policymaking was characterized less by commitment to any single assetization instrument than by the construction of a broad governance foundation. The coexistence of multiple themes at comparable intensity suggests a deliberate preference for institutional preparation over rapid marketization, reflecting an effort to stabilize security, coordination, and regulatory conditions before advancing more specific value-realization mechanisms. Although data was formally recognized as a factor of production during this period, the policy discourse remained cautious, prioritizing administrative coordination, regulatory compatibility, and systemic risk containment over explicit value realization. This cautious orientation is consistent with the institutional context in which medical data governance is embedded. Unlike conventional economic assets, medical data is closely linked to public welfare, professional ethics, and legal accountability. As a result, early national policies appear to have treated assetization as a long-term objective contingent upon the stabilization of governance prerequisites, including security standards, interdepartmental coordination mechanisms, and regulatory clarity. The absence of a dominant theme in this stage should therefore not be interpreted as policy indecision, but rather as a deliberate strategy to avoid premature commitment to narrowly defined market instruments.

The temporary rise of valuation discourse in 2023–2024 is best understood as a phase of agenda activation rather than as evidence of stable institutional consolidation. It signaled a willingness at the central level to explore capitalization, accounting recognition, and pricing mechanisms, but its subsequent retreat indicates that policy ambition had moved ahead of the institutional conditions required for sustained implementation. This surge coincided with broader national initiatives promoting data as a core production factor, and it played an important role in legitimizing experimentation by public institutions and market actors. However, the DTM results also show that this valuation-oriented emphasis was short-lived. By 2025, the intensity of valuation discourse declined markedly, while themes related to public data authorized operation and trusted infrastructure expanded to become the central focus of national policy attention. This inversion suggests a corrective adjustment rather than a policy reversal. The retreat of valuation from the center of the agenda reflects the practical constraints encountered when attempting to operationalize pricing mechanisms in the absence of stable authorization frameworks and enforceable operational boundaries. Valuation presupposes clearly defined rights, predictable usage scenarios, and reliable compliance structures. Where these conditions remain underdeveloped, valuation discourse risks outpacing institutional capacity. The subsequent re-centering of policy attention on authorized operation indicates a recognition that sustainable data assetization depends less on abstract pricing models than on the establishment of legally and administratively viable modes of data use.

Methodological diagnostics from the national DTM analysis further reinforce the need for cautious interpretation of short-term agenda surges. Evaluation indicators point to heterogeneity in topic coherence and temporal stability, particularly for emergent themes such as valuation. This suggests that sudden increases in topic intensity may reflect concentrated policy signaling or rhetorical emphasis rather than consolidated institutional change. Within this constraint, the observed pattern still supports an interpretation of adaptive governance, in which national policymakers test ambitious narratives, assess implementation frictions, and recalibrate priorities accordingly.

Taken together, the national policy trajectory reveals an iterative governance logic characterized by phased exploration and strategic correction. Rather than advancing data assetization through a single, cumulative pathway, the central government repeatedly reassesses feasibility under evolving regulatory and technological conditions. The eventual prioritization of authorized operation and infrastructural safeguards underscores a preference for controllable and scalable governance instruments. In this sense, non-linearity is not a sign of inconsistency, but a structural feature of policy learning in a domain where economic innovation is tightly coupled with public accountability and risk management.

Placed in the broader literature on digital health data governance, this pattern is not entirely unexpected. Prior studies have shown that China’s health and medical big data policies have long combined development, application, protection, and governance objectives, while policy refinement still depends on more specific operational rules and stronger diffusion of effective policy designs (4). Related policy-text analyses likewise suggest that Chinese data-element and smart-city policies do not advance in a straightforward linear sequence; rather, they undergo stage-based shifts from infrastructure and development priorities toward governance adjustment and data management (19, 20). Against this background, the non-linear trajectory identified here can be understood as a sector-specific manifestation of a broader governance logic in which institutional readiness, regulatory calibration, and administrative capacity shape the timing and scope of data valorization.

### 4.2 Central–local divergence as a functional division of policy labor

A consistent message emerging from the combined national and provincial evidence is that central–local divergence in medical data assetization should be understood less as fragmentation than as a functional division of policy labor. National policies are primarily oriented toward defining legitimacy conditions, regulatory boundaries, and scalable operational frameworks, whereas provincial policies tend to translate these broad principles into procedural arrangements and market-facing infrastructures. This differentiation is not unique to data governance, but it becomes especially visible in the medical domain, where commercialization pressures intersect with heightened ethical, privacy, and public-sector accountability requirements.

At the national level, the policy trajectory points to a governance logic centered on boundary-setting and risk containment. What matters analytically is not simply that priorities shifted, but that the shift moved toward instruments—authorized operation, trusted infrastructure, and security capacity—that are more compatible with nationwide legitimacy and controllability in a sensitive public-sector domain. This pattern indicates that central policymaking is less concerned with prescribing how local actors should transact in practice than with establishing nationally legitimate modes of operation—clarifying who may use data, under what authorization, with what responsibilities, and within which security constraints. In other words, the center’s role is to provide the institutional “rails” that prevent market-oriented experimentation from eroding public trust or triggering systemic compliance risks.

Provincial policies, by contrast, reflect a more execution-oriented logic. Rather than remaining at the level of strategic discourse, local governments moved toward mechanisms that could be embedded in administrative routines—registration, rights confirmation, platform governance, and standardized circulation procedures—thereby translating broad national principles into operational arrangements. The steady strengthening of trading and circulation language further suggests that local governments are actively constructing market infrastructures—data exchanges, service platforms, intermediary ecosystems—even while national-level discourse on valuation fluctuates. This evolution highlights an important feature of provincial governance: it is less dependent on resolving high-level conceptual debates about ownership and more focused on establishing workable procedures that can be audited, supervised, and operationalized.

This central–local contrast also helps explain why “registration” becomes a persistent local focal point. In the absence of a fully unified national property-rights framework for data, provinces appear to rely on registration-based mechanisms as institutional substitutes. Such mechanisms do not necessarily resolve ownership controversies in a theoretical sense, but they can formalize operational rights, delineate responsibility chains, and create administrative evidence for subsequent circulation activities. Registration, in this context, functions as a governance technology: it makes data rights legible to the state, reduces ambiguity for platform operators and intermediaries, and provides a basis for compliance review. The emergence of “authorized operation” and “registration” in provincial topic structures thus signals not merely bureaucratic formality, but an attempt to render data circulation governable within existing administrative capacities.

At the same time, provincial experimentation introduces heterogeneity in implementation pathways. Local governments differ in digital infrastructure maturity, industrial demand for medical data, and institutional capacity to manage privacy and security compliance. As a result, provinces may prioritize different combinations of platforms, intermediaries, and procedural controls. This heterogeneity is not necessarily problematic in early-stage governance; it can generate policy learning and reveal workable models. However, it also creates risks of incompatibility across regions, particularly if registration systems, platform standards, or transaction rules are not interoperable. In the medical domain, where cross-institutional data linkage is often a prerequisite for high-value applications (such as multi-center clinical research or AI model training), fragmentation in local rules may constrain scale effects and raise transaction costs.

The evaluation diagnostics from the provincial DTM analysis provide an additional nuance for interpretation. While overall temporal smoothness is relatively high, the topic most directly associated with registration and authorized operation exhibits lower temporal coherence, indicating that its semantic content may evolve across time slices as provinces refine operational details. This does not undermine the substantive trend, but it suggests that local governments are not simply repeating a fixed template; rather, they are iteratively adjusting administrative procedures and governance language in response to policy signals and implementation feedback. Such dynamics are consistent with an incremental and pragmatic style of local governance that prioritizes operability over conceptual closure.

Taken together, the central–local divergence observed in this study reflects a governance arrangement in which strategic design and systemic risk control are centralized, while procedural innovation and market testing are decentralized. The center establishes permissible boundaries and legitimacy conditions, and provinces develop concrete mechanisms—registration workflows, circulation platforms, and compliance routines—that make assetization practically achievable. This division of labor helps explain why national discourse may appear to “pivot” across themes while local agendas display persistent attention to registration and circulation: they operate at different layers of the policy system, with different constraints and responsibilities. In this sense, divergence is not a sign of inconsistency but an institutional feature that enables policy ambition to coexist with the public accountability demands of medical data governance.

This interpretation also resonates with recent studies on public-data and health-data governance in China. Research on provincial public data management measures shows that local rules tend to rely heavily on responsibility allocation and operational support arrangements, yet still exhibit weaknesses in security safeguards and supervision. This suggests that local policy innovation often advances through procedural instruments rather than through fully settled property-rights frameworks (21). At the same time, quantitative analyses of health and medical big data policies identify marked variation in policy effectiveness and tool combinations across central and provincial texts, indicating that local adaptation remains structurally important even within a common national agenda (4). The present findings extend this line of scholarship by showing that, in the specific context of medical data assetization, registration and platform-based circulation are better understood not as routine administrative procedures, but as governance technologies through which provinces translate national principles into workable arrangements under conditions of legal and institutional incompleteness.

### 4.3 The valuation bottleneck and the sequencing logic of medical data assetization

Valuation occupies a prominent but unstable position in the policy discourse on medical data assetization, and this instability is analytically significant. Rather than indicating a simple fluctuation in policy attention, it points to a structural bottleneck: valuation is politically attractive as a reform signal, but far more difficult to institutionalize than mechanisms such as authorized operation, registration, or infrastructural governance.

From an institutional perspective, valuation represents one of the most demanding stages of assetization. Unlike registration or authorized operation, which can be embedded within existing administrative routines, valuation requires the alignment of legal rights, accounting standards, and market expectations. In the medical data context, these conditions are particularly difficult to satisfy. Data have non-rival characteristics as knowledge-like assets (29), and subject to stringent ethical and regulatory constraints. As a result, the prerequisites for credible and enforceable valuation are significantly higher than those for procedural governance mechanisms.

The policy trajectory reflects these constraints in a revealing way. The prominence of valuation discourse can be read as a strategic signal intended to coordinate expectations around pricing, accounting, and value realization; its later decline, however, suggests that such signaling could not by itself compensate for unresolved problems of authorization, rights clarity, and operational feasibility. Without stable authorization frameworks and clearly delineated operational rights, valuation risks becoming detached from actual usage scenarios, undermining its credibility as a governance instrument.

Accounting practices further illustrate this bottleneck. Existing valuation approaches for public-sector data assets tend to rely on cost-based methods (30), which are compatible with conservative public asset management principles but offer limited insight into potential downstream value. Income-based or market-based methods (30), while theoretically appealing, face substantial obstacles in the medical domain. Revenue streams from data-driven applications are often indirect, uncertain, and distributed across multiple actors, making it difficult to attribute value to the underlying data asset itself. The absence of standardized benchmarks exacerbates this problem, resulting in wide valuation ranges that are difficult to justify in regulatory or audit contexts.

Beyond technical accounting issues, compliance costs impose an additional constraint on valuation. Medical data governance requires extensive investment in privacy protection, security infrastructure, and regulatory oversight, including de-identification, access control, and audit mechanisms. These costs are not always visible in valuation models but have a direct impact on the economic viability of data transactions. When such costs are internalized, the gap between nominal asset value and realizable transaction value becomes apparent, reducing incentives for both data holders and potential users to engage in pricing-based exchanges.

Property-rights ambiguity further compounds the valuation bottleneck. In public healthcare systems, data-related rights are distributed among patients, healthcare professionals, institutions, and regulators. While authorized operation frameworks can clarify who may use data under specified conditions, they do not automatically resolve deeper questions of ownership or residual claims. Valuation, however, presupposes a degree of rights clarity that allows prices to function as meaningful signals. The empirical finding that registration and authorized operation intensify before valuation stabilizes suggests that policymakers recognize this dependency and are sequencing reforms accordingly.

Provincial policy trajectories reinforce this interpretation. Rather than prioritizing valuation as an immediate objective, provinces place sustained emphasis on registration, circulation platforms, and procedural compliance. Trading and circulation themes strengthen even in the absence of stable pricing standards, indicating that local governments are advancing market infrastructures while postponing full-scale valuation. This “build-while-regulating” approach reflects a pragmatic assessment of feasibility: circulation can occur under regulated conditions without requiring precise asset pricing, whereas valuation without circulation lacks practical grounding.

Methodological diagnostics from the DTM analysis also caution against over interpreting short-term valuation peaks as evidence of institutional consolidation. Topic stability and coherence indicators suggest that valuation-related discourse is particularly sensitive to concentrated policy signaling. This reinforces the interpretation of valuation as a high-salience but low-institutionalization agenda, whose prominence fluctuates in response to strategic emphasis rather than sustained implementation capacity.

Taken together, these findings point to a sequencing logic in China’s approach to medical data assetization. Valuation is not abandoned, but deferred. It functions as a horizon objective that shapes long-term expectations while yielding, in the near term, to more controllable governance instruments such as authorized operation, registration, and secure infrastructure. This sequencing reflects a cautious balance between innovation and accountability. By postponing the financialization of medical data until institutional prerequisites are more fully developed, policymakers seek to preserve public trust and regulatory legitimacy while gradually expanding the scope for market-oriented data use.

In this sense, the valuation bottleneck should not be understood as a policy failure. Rather, it is an indicator of institutional self-restraint in a domain where economic experimentation is inseparable from ethical responsibility and systemic risk. The episodic prominence of valuation discourse thus serves as a strategic signal rather than a definitive endpoint, anchoring future reform trajectories while allowing governance capacity to mature.

This reading is also supported by the emerging literature on data valuation and medical data commercialization. Veldkamp argues that data differ from conventional assets because their value is highly context-dependent, varies substantially across users, and is difficult to capture through standard asset-pricing tools, which helps explain why valuation discourse may rise rapidly at the rhetorical level before stable pricing institutions take shape (23). Chinese studies similarly note that cost-based approaches remain the most feasible in current practice, whereas income-based and market-based methods face substantial constraints arising from uncertain future returns, inactive comparable markets, and weak benchmark systems (3, 31). Recent work on trusted circulation further suggests that sustainable data markets depend less on abstract pricing claims than on credible institutional arrangements capable of reducing transaction frictions and supporting compliant in-market exchange (32). This is consistent with our finding that valuation has not disappeared from the policy agenda, but is being sequenced behind authorized operation, circulation governance, and infrastructure building.

### 4.4 Global implications and governance recommendations for medical data assetization

The evidence presented in this study suggests that medical data assetization in China is progressing through a governance pathway in which operability precedes full financialization. The national trajectory shows a clear corrective movement toward authorized operation and trusted infrastructure after a short valuation-oriented surge, while provincial trajectories exhibit persistent attention to registration and circulation mechanisms. Taken together, these findings imply that the near-term policy challenge is less about designing ever more sophisticated pricing models and more about consolidating the institutional and technical conditions that make lawful, accountable, and scalable circulation possible. This section outlines several implications that follow directly from that sequencing logic.

A first implication concerns the institutional centrality of authorized operation as the national “hinge” mechanism. The national pivot toward authorized operation indicates that policymakers treat it as the primary solution for reconciling two competing demands: enabling data use for innovation and safeguarding public interest in a highly sensitive domain. If authorized operation is to function as more than a policy slogan, it must be made auditable and enforceable in practice. This requires clearer operational specifications—who qualifies as an operator, what constitutes permissible use, and how responsibility is assigned across data providers, operators, and downstream users. Importantly, authorized operation should not be framed as a single administrative approval step; rather, it needs to be institutionalized as an ongoing governance arrangement supported by monitoring, traceability, and accountability mechanisms. Without this, local experimentation risks turning into fragmented and difficult-to-supervise practices that undermine the very legitimacy authorized operation is meant to secure.

Second, the provincial emphasis on registration highlights the practical demand for a rights-confirmation technology that can be implemented under existing administrative capacity. Registration appears to function as a policy instrument that makes rights legible and responsibilities traceable, even when deeper ownership questions remain contested. This has two governance implications. On the one hand, policymakers can leverage registration as a pragmatic substitute that enables circulation to begin under controlled conditions. On the other hand, registration systems must be designed with interoperability in mind. If registration rules, certificates, or metadata standards vary widely across provinces, transaction costs will rise, cross-regional circulation will be impeded, and “local markets” will remain small and segmented. A realistic policy goal, therefore, is not immediate national uniformity, but the gradual construction of minimum common standards—core registration fields, shared classification schemes, and mutually recognizable compliance labels—that allow different provincial systems to communicate.

Third, the sustained strengthening of circulation and trading themes at the provincial level suggests that market infrastructure is being built even when valuation remains uncertain. This “build-while-regulating” pattern can be productive if it is coupled with constraints that prevent premature commercialization from generating compliance failures. Policy design should therefore distinguish between (a) circulation mechanisms that primarily facilitate regulated access for public-interest purposes (research, public health, quality improvement) and (b) market-facing transactions aimed at commercial value extraction. These two types of circulation require different governance intensity. The former can often be scaled through standardized access protocols and secure computation arrangements; the latter requires more stringent rules on purpose limitation, benefit distribution, and accountability. Blurring these categories increases the risk that high-sensitivity medical data is drawn into commercial exchanges without adequate safeguards, which would likely provoke backlash and policy retrenchment.

Fourth, the observed valuation bottleneck implies that valuation should be treated as a staged capacity-building project rather than an immediate regulatory deliverable. The national trajectory suggests that valuation discourse can be useful for agenda-setting and expectation alignment, but it becomes fragile when institutional preconditions are incomplete. A more feasible approach is to build valuation capability through bounded pilots that link pricing experiments to well-defined use cases and measurable governance outputs. Instead of attempting to assign an abstract “asset price” to medical data as a whole, pilots can focus on context-specific valuation questions: pricing secure access for narrowly defined research queries, estimating value contributions within multi-party AI development pipelines, or benchmarking compliance costs and their impact on realizable value. Over time, such pilots can generate reference points and reduce uncertainty, allowing valuation to evolve from rhetorical emphasis into institutional practice.

Fifth, the temporal and spatial patterns documented here highlight a broader coordination challenge: policy diffusion is staged, and regional heterogeneity is structural. The synchronized surge of provincial policy activity after key national frameworks became clearer suggests that local governments respond strongly once boundaries are specified. At the same time, regional differences in capacity, industrial demand, and governance maturity mean that a one-size-fits-all implementation mandate is unlikely to succeed. A more realistic national strategy is to govern through layered coordination: defining nationally consistent bottom lines (security, accountability, public interest protections), while allowing provinces to innovate in procedural design and platform construction within those boundaries. The key is to ensure that innovation remains compatible with future harmonization. This is where national-level standard-setting—technical interfaces, compliance audit criteria, and cross-regional recognition mechanisms—becomes essential. Without it, local innovation may produce a patchwork of rules that cannot scale.

Finally, these implications also speak to the role of public healthcare institutions as the principal data holders. The policy shift toward authorized operation and registration inevitably changes how public institutions are expected to behave: from passive custodians of clinical records to active participants in governance and circulation. Yet public healthcare institutions face asymmetric risks—legal liability, reputational harm, and ethical scrutiny—while the benefits of data use may be realized elsewhere in the ecosystem. Policy frameworks should therefore address incentive compatibility. This includes clarifying responsibility boundaries for institutions that provide data under authorized operation, specifying acceptable benefit-sharing mechanisms, and supporting institutional capacity building in compliance, security, and governance. Without such measures, hospitals may rationally adopt overly conservative strategies that inhibit legitimate data use, leaving policy ambition unrealized.

In sum, the results suggest a pragmatic governance roadmap: consolidate authorized operation as a credible institutional anchor; treat registration as a scalable governance technology while preventing fragmentation through minimum interoperability standards; expand circulation in differentiated modes that match risk profiles; and develop valuation through staged, use-case-based pilots rather than abstract universal pricing. This sequencing respects the high-stakes nature of medical data while preserving space for innovation. It also offers a more realistic basis for aligning national strategic objectives with provincial implementation dynamics, reducing the likelihood that policy agendas oscillate between ambitious financialization narratives and corrective retrenchment driven by compliance shocks.

The policy recommendations advanced here are also broadly in line with recent research on medical data value release, data trading policy, and public-data governance. Value-chain and simulation studies suggest that the release of medical data value depends not only on the participation of healthcare institutions, but also on active governmental involvement, effective market regulation, risk control, and trust maintenance (11). Policy analyses of medical data trading further indicate that future reform needs to move beyond macro-level guidance toward more concrete institutional support, including operational safety rules, incentive-compatible benefit-sharing, third-party service ecosystems, and infrastructure capacity (3). Meanwhile, studies of provincial public data rules underscore the importance of strengthening security guarantees, supervision, and interoperable support mechanisms in order to prevent fragmentation during local implementation (21). The governance roadmap proposed in this study should therefore be read not simply as an interpretation of our own empirical results, but as a synthesis increasingly supported by adjacent evidence from health data policy, public data governance, and data-element market research.

## 5 Conclusion

This study examined the evolution of China’s medical data assetization policies through a combined application of Latent Dirichlet Allocation (LDA) and Dynamic Topic Modeling (DTM) to national and provincial policy documents. By integrating static thematic structures with dynamic temporal patterns, the analysis provides an empirical account of how policy priorities surrounding medical data have developed and adjusted over time within China’s public healthcare system.

At the national level, the results reveal a non-linear policy trajectory characterized by adjustment rather than continuous progression. Early policies displayed a relatively balanced emphasis on digital infrastructure, governance coordination, market mechanisms, and data security, reflecting a cautious approach to system-building. Although valuation-related discourse rose sharply in the mid-2020s, this emphasis proved unstable. In the most recent phase, national policy attention shifted toward authorized operation and trusted infrastructure, indicating a recalibration away from premature financialization. This pattern suggests a sequencing logic in which authorization, security, and operational feasibility are treated as prerequisites for sustainable data assetization, rather than valuation being pursued as an immediate governing core.

Provincial policies exhibit a complementary but distinct trajectory. While early local initiatives were closely linked to digital government and industrial development agendas, provincial priorities increasingly converged on registration, circulation mechanisms, and platform-based implementation. Rather than focusing on abstract questions of ownership or pricing, local governments emphasized procedural arrangements that could be embedded within existing administrative systems. Registration and authorized operation thus function as key instruments enabling regulated data circulation at the local level, even in the absence of a unified national property-rights framework.

Taken together, the findings highlight a functional differentiation between central and local governance. The national level primarily defines strategic direction, legitimacy boundaries, and scalable institutional frameworks, whereas provincial governments translate these principles into operational mechanisms and market infrastructures. This division of labor helps explain why national policy agendas may shift across themes while local policies display continuity in implementation-oriented instruments. Such divergence reflects institutional design rather than policy inconsistency.

More broadly, the study suggests that medical data assetization in China is advancing through a cautious and adaptive governance process. Valuation plays an important signaling role but remains constrained by unresolved issues related to rights clarity, compliance costs, and operational enforceability. In the current stage, consolidating authorized operation frameworks, strengthening registration systems, and ensuring secure circulation appear to be the most critical steps toward realizing the economic potential of medical data while maintaining public trust.

The contribution of this study is threefold. Theoretically, it advances the understanding of medical data assetization as a process of institutional change embedded in multi-level governance, and shows that its policy evolution follows a sequencing logic in which authorization, registration, and secure circulation precede stable valuation. Empirically, it provides systematic evidence of a functional differentiation between central and provincial governance, in which the national level defines strategic boundaries and legitimacy conditions while provincial governments translate these principles into operational mechanisms. Practically, the findings offer a policy-relevant framework for strengthening authorized operation, registration systems, differentiated circulation arrangements, and staged valuation pilots in the governance of medical data. Although the analysis is limited to policy texts and does not assess market outcomes, it offers a systematic foundation for future research on the interaction between institutional design and data-driven innovation in healthcare.

## 6 Limitations and future directions

This study focuses on the textual analysis of policy documents and, as such, does not directly evaluate the economic outcomes associated with medical data assetization. While topic modeling offers a systematic way to capture policy priorities and their evolution over time, it cannot by itself determine whether policy signals have translated into measurable market activity, such as transaction volumes, pricing outcomes, or organizational performance. Accordingly, the findings should be interpreted as evidence of institutional and discursive dynamics rather than as direct indicators of implementation effectiveness or economic performance.

Another limitation concerns the scope of policy texts analyzed. Although the corpus covers a wide range of national and provincial policy documents, it does not capture informal regulatory practices, local implementation guidelines, or negotiations between public institutions and market actors that may shape data circulation in practice. These processes can be particularly influential in the medical domain, where compliance considerations and risk perceptions often affect behavior beyond what is formally articulated in policy documents.

Future research could address these limitations in several ways. First, combining policy analysis with quantitative evidence—such as data transaction records, platform activity, or organizational case data—would allow for a more direct assessment of how policy evolution interacts with market outcomes. Second, in-depth case studies of selected provinces or pilot programs could provide richer insight into how authorized operation, registration, and circulation mechanisms are implemented on the ground, and how local institutional capacity conditions their effectiveness. Finally, extending the analytical framework to a comparative context would help clarify which aspects of China’s experience are shaped by its specific governance structure and which may offer more general lessons for other health systems seeking to balance data-driven innovation with public accountability (33). Taken together, these directions would help move future research beyond policy texts alone and toward a more integrated understanding of how institutional design, implementation processes, and market outcomes interact in the assetization of medical data.

## Data Availability

The original contributions presented in the study are included in the article/Supplementary material, further inquiries can be directed to the corresponding author.
